# Supplementary material for: New Insights into the Implication of Epigenetic Alterations in the EMT of Triple Negative Breast Cancer
Source: Cancers (Basel). 2019 Apr 18;11(4):559. doi: 10.3390/cancers11040559 (PMC6521131; doi:10.3390/cancers11040559)
Supplement: Supplementary file 1 [file cancers-11-00559-s001.zip › Interactive_Network/index.html]

OII Network Visualisation Example


 


:   More about this visualisation

## Legend:

Interactive network of epigenetic factors driving EMT in Triple Negative Breast Cancers


Factors are grouped and colored based on their nature/function (details below in the Function Selector).

The graphic is oriented from the periphery to the center. Green edges figure activation, as red ones figure repression.

## Search:

## Function Selector:

Select Function

Return to the full network

Information Pane

Connections:

OII


JISC
