# Supplementary material for: New Insights into the Implication of Epigenetic Alterations in the EMT of Triple Negative Breast Cancer
Source: Cancers (Basel). 2019 Apr 18;11(4):559. doi: 10.3390/cancers11040559 (PMC6521131; doi:10.3390/cancers11040559)
Supplement: Supplementary file 1 [file cancers-11-00559-s001.zip › Interactive_Network/Legend Figure1.docx]

**Figure1: Interactive network of epigenetic factors driving EMT in Triple Negative Breast Cancers.**

Figure 1 assembles in a unique network the latest findings regarding the implication of the four major classes of epigenetic factors (lncRNA, miRNA, and histone or DNA modification) in the regulation of EMT in TNBC. Main EMT inducing pathways (TGFß, Wnt, etc), transcription factors (Zeb, Snail, etc), and functional markers (E-cadherin, Vimentin, etc) affected by the deregulation of these epigenetic modifiers are placed. Target genes of DNA methylation and histones modifications as well as their downstream targets are posted.

Molecular interactions between factors are drawn by centripetal coloured edges. The graphic is oriented from the periphery to the center. Green edges figure activation, as red ones figure repression.

Higher number of connections for a node/factor signs higher level of knowledge. A node connected directly to EMT means a lack of evidence about its molecular role.

Factors are grouped and colored based on their nature/function (details below in the Function Selector).
