# Supplementary figures and images for: New Insights into the Implication of Epigenetic Alterations in the EMT of Triple Negative Breast Cancer
Source: Cancers (Basel). 2019 Apr 18;11(4):559. doi: 10.3390/cancers11040559 (PMC6521131; doi:10.3390/cancers11040559)

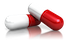

Supplement: Supplementary file 1 [file cancers-11-00559-s001.zip › Interactive_Network/images/2pills.png]

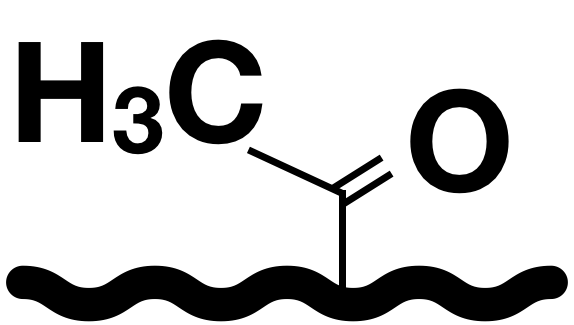

Supplement: Supplementary file 1 [file cancers-11-00559-s001.zip › Interactive_Network/images/Acethylation.png]

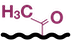

Supplement: Supplementary file 1 [file cancers-11-00559-s001.zip › Interactive_Network/images/Acethylation_rose.png]

Supplement: Supplementary file 1 [file cancers-11-00559-s001.zip › Interactive_Network/images/blank.gif]

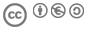

Supplement: Supplementary file 1 [file cancers-11-00559-s001.zip › Interactive_Network/images/CC.png]

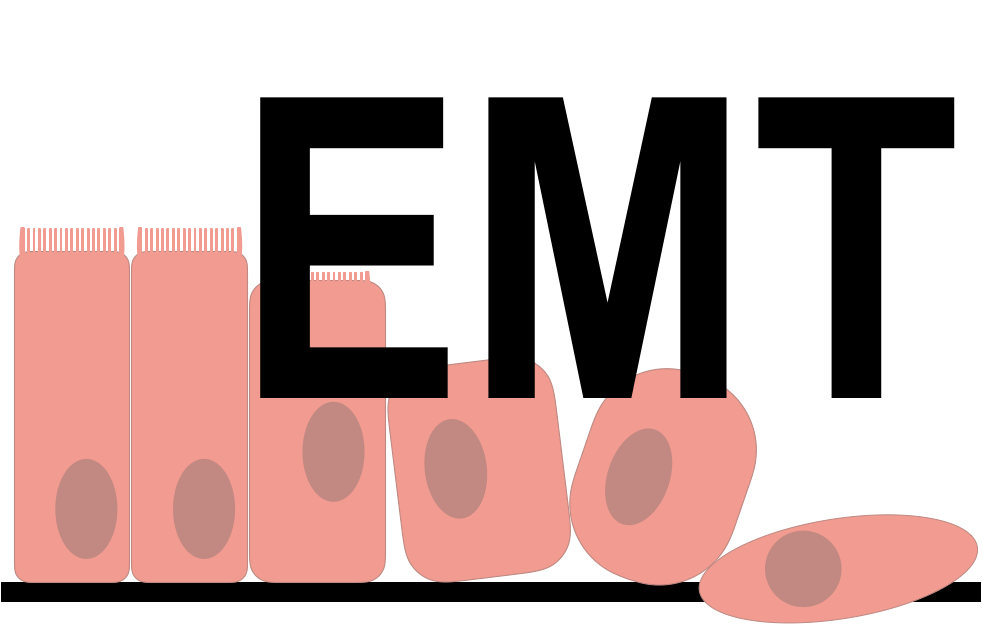

Supplement: Supplementary file 1 [file cancers-11-00559-s001.zip › Interactive_Network/images/EMT.png]

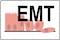

Supplement: Supplementary file 1 [file cancers-11-00559-s001.zip › Interactive_Network/images/EMT_blanc.png]

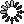

Supplement: Supplementary file 1 [file cancers-11-00559-s001.zip › Interactive_Network/images/fancybox_loading.gif]

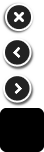

Supplement: Supplementary file 1 [file cancers-11-00559-s001.zip › Interactive_Network/images/fancybox_sprite.png]

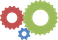

Supplement: Supplementary file 1 [file cancers-11-00559-s001.zip › Interactive_Network/images/Gears.png]

## Slide 1
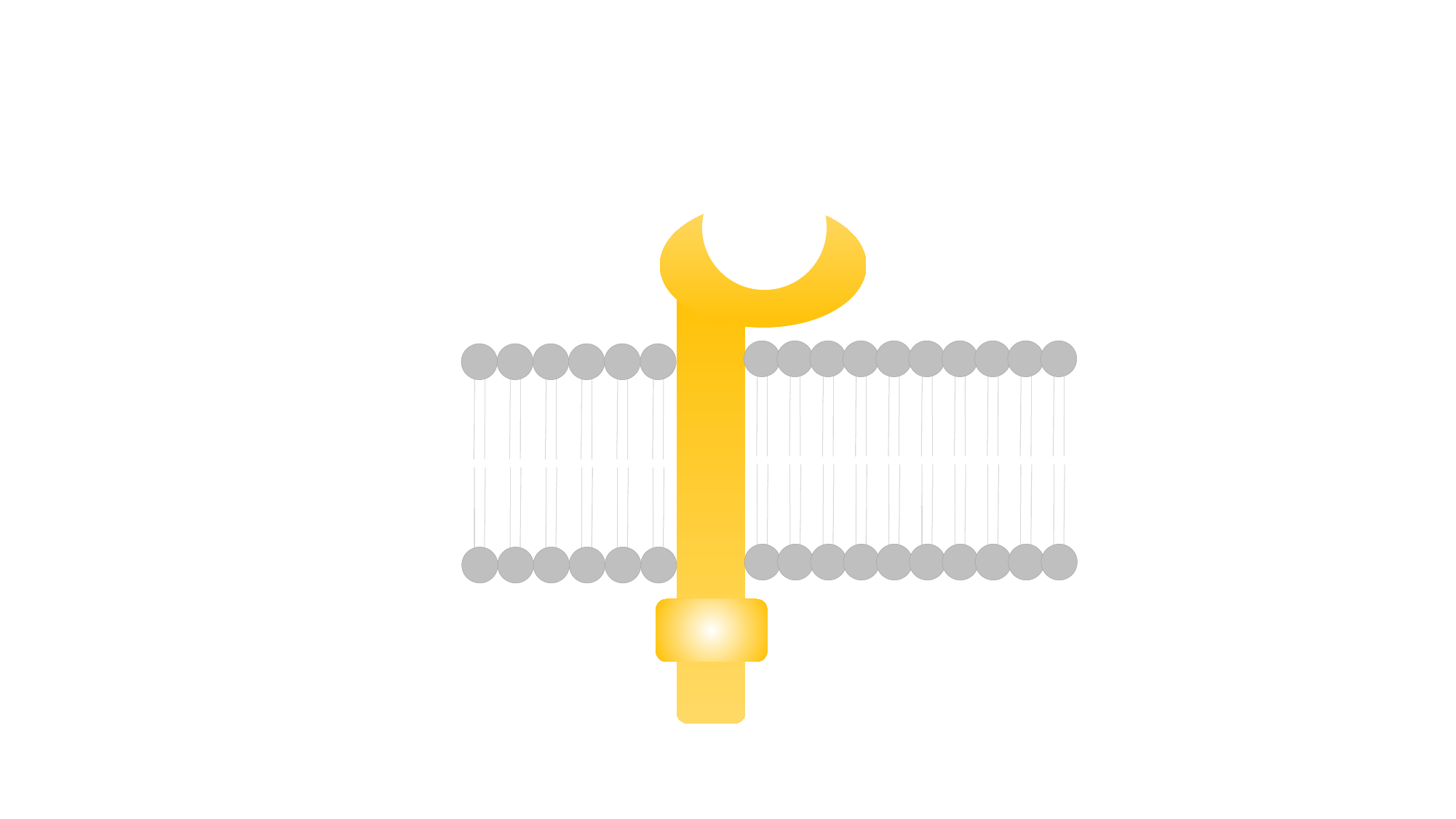

Supplement: Supplementary file 1 [file cancers-11-00559-s001.zip › Interactive_Network/images/Images Network.pptx]

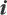

Supplement: Supplementary file 1 [file cancers-11-00559-s001.zip › Interactive_Network/images/info.png]

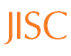

Supplement: Supplementary file 1 [file cancers-11-00559-s001.zip › Interactive_Network/images/jisc-logo-small.png]

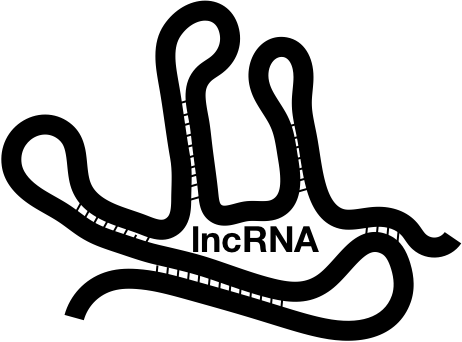

Supplement: Supplementary file 1 [file cancers-11-00559-s001.zip › Interactive_Network/images/lncRNA.png]

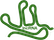

Supplement: Supplementary file 1 [file cancers-11-00559-s001.zip › Interactive_Network/images/lncRNA_vert.png]

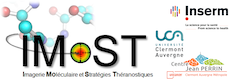

Supplement: Supplementary file 1 [file cancers-11-00559-s001.zip › Interactive_Network/images/Logos.png]

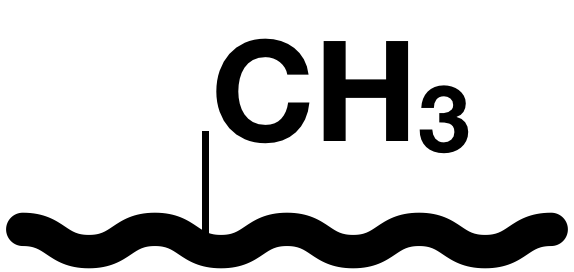

Supplement: Supplementary file 1 [file cancers-11-00559-s001.zip › Interactive_Network/images/Methylation.png]

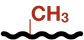

Supplement: Supplementary file 1 [file cancers-11-00559-s001.zip › Interactive_Network/images/Methylation_rouge.png]

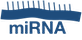

Supplement: Supplementary file 1 [file cancers-11-00559-s001.zip › Interactive_Network/images/miRNA_bleu.png]

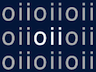

Supplement: Supplementary file 1 [file cancers-11-00559-s001.zip › Interactive_Network/images/oii.png]

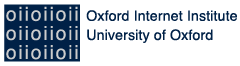

Supplement: Supplementary file 1 [file cancers-11-00559-s001.zip › Interactive_Network/images/oii_brand.png]

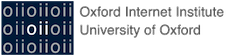

Supplement: Supplementary file 1 [file cancers-11-00559-s001.zip › Interactive_Network/images/oii_text.png]

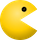

Supplement: Supplementary file 1 [file cancers-11-00559-s001.zip › Interactive_Network/images/Pacman.png]

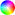

Supplement: Supplementary file 1 [file cancers-11-00559-s001.zip › Interactive_Network/images/rainbow.png]

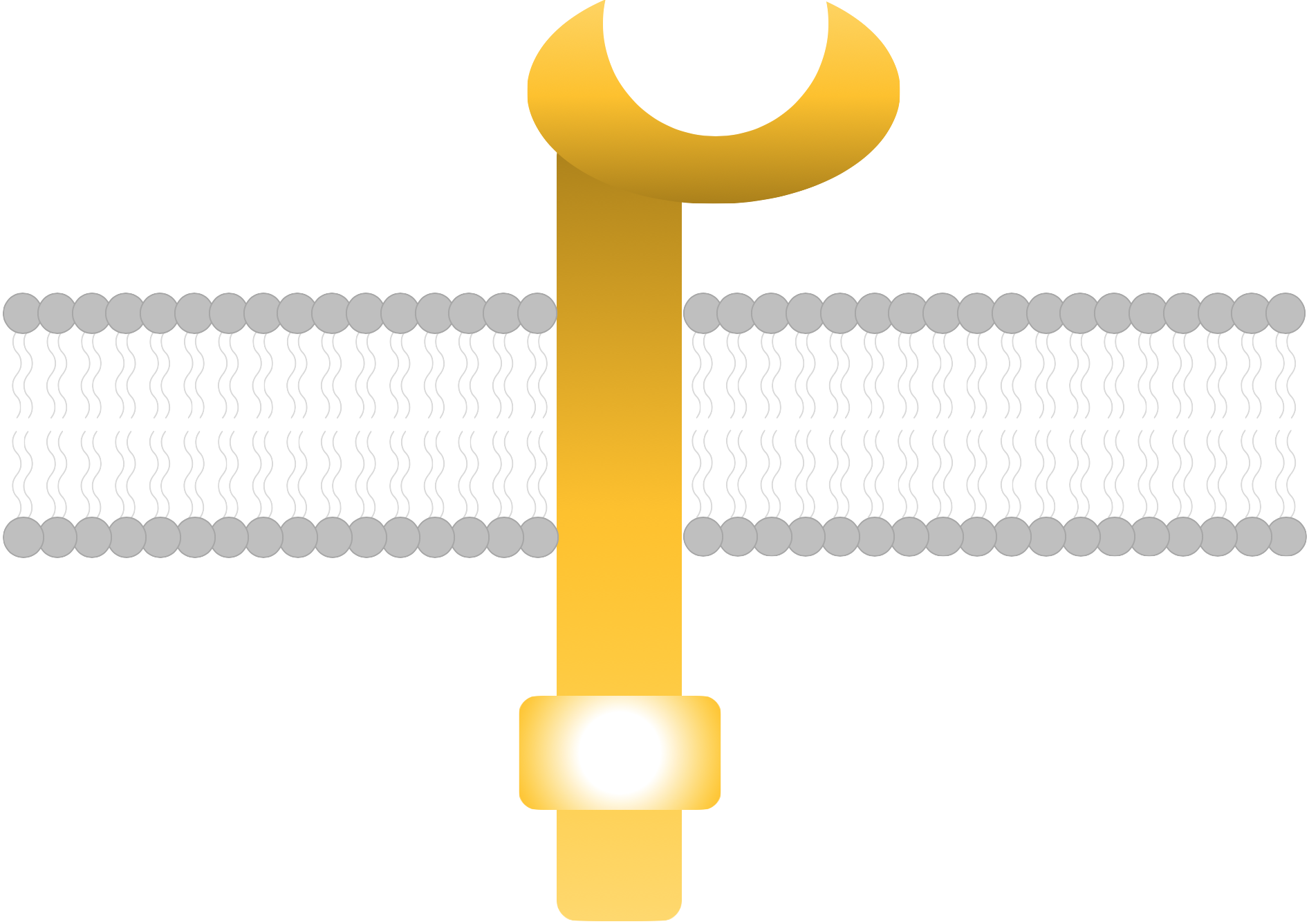

Supplement: Supplementary file 1 [file cancers-11-00559-s001.zip › Interactive_Network/images/Receptor_orange 1.png]

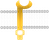

Supplement: Supplementary file 1 [file cancers-11-00559-s001.zip › Interactive_Network/images/Receptor_orange.png]

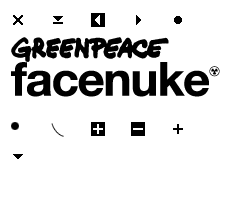

Supplement: Supplementary file 1 [file cancers-11-00559-s001.zip › Interactive_Network/images/sprite.png]

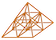

Supplement: Supplementary file 1 [file cancers-11-00559-s001.zip › Interactive_Network/images/Structure.png]

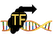

Supplement: Supplementary file 1 [file cancers-11-00559-s001.zip › Interactive_Network/images/Transcription_Factor.png]

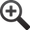

Supplement: Supplementary file 1 [file cancers-11-00559-s001.zip › Interactive_Network/images/zoom_in.png]

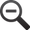

Supplement: Supplementary file 1 [file cancers-11-00559-s001.zip › Interactive_Network/images/zoom_out.png]

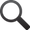

Supplement: Supplementary file 1 [file cancers-11-00559-s001.zip › Interactive_Network/images/zoom_reset.png]
